# Anomalous luminescence phenomena of indium-doped ZnO nanostructures grown on Si substrates by the hydrothermal method

**DOI:** 10.1186/1556-276X-7-270

**Published:** 2012-05-30

**Authors:** Jen-Cheng Wang, Fang-Ching Cheng, Yu-Ting Liang, Hung-Ing Chen, Chung-Yuan Tsai, Chia-Hui Fang, Tzer-En Nee

**Affiliations:** 1 Graduate Institute of Electro-Optical Engineering and Department of Electronic Engineering, Chang Gung University, Kwei-Shan, Tao-Yuan, 333, Taiwan

**Keywords:** Zinc oxide (ZnO), Nanostructure, Hydrothermal method

## Abstract

In recent years, zinc oxide (ZnO) has become one of the most popular research materials due to its unique properties and various applications. ZnO is an intrinsic semiconductor, with a wide bandgap (3.37 eV) and large exciton binding energy (60 meV) making it suitable for many optical applications. In this experiment, the simple hydrothermal method is used to grow indium-doped ZnO nanostructures on a silicon wafer, which are then annealed at different temperatures (400°C to 1,000°C) in an abundant oxygen atmosphere. This study discusses the surface structure and optical characteristic of ZnO nanomaterials. The structure of the ZnO nanostructures is analyzed by X-ray diffraction, the superficial state by scanning electron microscopy, and the optical measurements which are carried out using the temperature-dependent photoluminescence (PL) spectra. In this study, we discuss the broad peak energy of the yellow-orange emission which shows tendency towards a blueshift with the temperature increase in the PL spectra. This differs from other common semiconductors which have an increase in their peak energy of deep-level emission along with measurement temperature.

## Background

Zinc oxide (ZnO) is an important II-VI compound semiconductor which belongs to the group with hexagonal close packing and has a wurtzite structure with P6_3_mc symmetry. The lattice constants of ZnO are a = 3.2539 Å and c = 5.2098 Å with a perfect ratio of c/a close to 1.633. This structure has hexagonal symmetry but not a center which displays excellent piezoelectric properties. Moreover, ZnO is a semiconductor with a wide bandgap (3.3 eV) and high exciton binding energy (60 meV) [1] that is a marvelous property for optics. It is widely recommended for optoelectronics [2], sensors [3], and transistors [4] and has wide applications in transparent conducting films [5], rheostats [6], and photocatalysis devices [7].

Recently, many special ZnO nanostructures such as nanocastles, nanocombs, and nanocages have been synthesized through various methods. Photoluminescence (PL) studies of ZnO have been conducted for several decades. Although the ultraviolet [8], violet, green [9], orange [10], and near IR emissions [11] of this material have been researched systematically by many groups, their proper emission mechanisms are still under dispute, especially the defect-related emissions. Green emissions of ZnO were first proposed by Vanheusden [12] who used the V_0_^*^ single ionized oxygen defect and band-bending interrelation to explain the phenomenon. A series of annealing processes are carried out, and variations in the intensity of the green emissions with the temperature dependence and oxygen vacancies are observed. The results prove that intensity of the green emission is related to the content of V_0_^*^.

In this study, the ZnO microrods are fabricated by the hydrothermal method [13-15], but the samples (ZnO) produced by this process have many defects. The PL measurements of the samples show yellow-orange emissions caused by annealing in an oxygen environment; however this may cause the occurrence of many oxygen vacancies or interstitials in the ZnO. The intrinsic emission of ZnO is also called the near band edge with a wavelength of about 378 nm (UV emission) [8] and has strong defect emissions which are called deep-level emissions with a wavelength of about 617 nm (yellow-orange emission) [10]. The strong defect emissions also occasionally include visual violet and infrared light. The violet emissions can be attributed to the recombination of electrons and holes between the interstitial zinc (Zn_i_) shallow donor levels and the valence band [16,17]. Normally, the n-type dopants for ZnO comprise the three grouped elements such as indium [18], aluminum and gallium. Silver and lithium have been used for p-type doping [19]. In this work, the ZnO nanostructure is doped with indium. This technique and doping method can change the carrier concentration leading to more research applications. Additionally, In-doped ZnO nanostructures have been grown by the hydrothermal method. They are easy to grow because of the low pressure and temperature.

The intrinsic emission is a result of the combination of free electrons or exciton-exciton oscillation. In addition, defect emission refers to the oxygen ionization vacancies above the surface and the single ionization charge of ZnO which combines with the holes. The phenomenon arising from strong defect emissions compared to weak intrinsic emissions is due to free-exciton annihilation caused by collision or recombination with each other. The result also reduces the luminescent excitons, the luminescence efficiency, and peak intensity.

## Methods

This hydrothermal method [20], which involves heterogeneous nucleation in supersaturated solutions to grow nanocrystals on the surface, has many advantages including being an easy procedure to use, requiring a low temperature and pressure. The surface roughness obtained using a buffer layer to synthesize ZnO offers heterogeneous nucleation points. After the cleaning process, the wafer is placed in acetone, DI water, and isopropyl alcohol (IPA). Zinc acetate which has been dissolved in the alcohol is uniformly distributed onto the silicon substrate in order to increase the density. Subsequently, ZnO nanostructures grow in the combination of zinc aqueous solution blended with 28% ammonia (NH_3_). Finally, the samples are coated with indium acetate which has been dissolved in alcohol and then annealed in a high-temperature furnace. The heat allows the indium ions to penetrate the ZnO nanostructures.

In this study, we analyze the structure and superficial state of ZnO by high-resolution X-ray diffraction (HR-XRD), and field-emission scanning electron microscopy (FE-SEM). ZnO optical measurements are utilized to survey the temperature-dependent PL spectra. The eight samples include non-annealing and annealing at temperatures from 400°C to 1,000°C. The temperature measurement range is from 20 to 300K, with a measuring point every 20K. We discuss the yellow-orange emission caused by excess oxygen vacancies in ZnO. For realizing the temperature dependence of PL peak energy of the samples, we use the Gaussian distribution to fit the data. Consequently, we prove that the dependence of the peak energy tends to vary with the crystal size as shown by the roughly calculated values of the full width at half maximum (FWHM).

## Results and discussion

**Figure 1:**
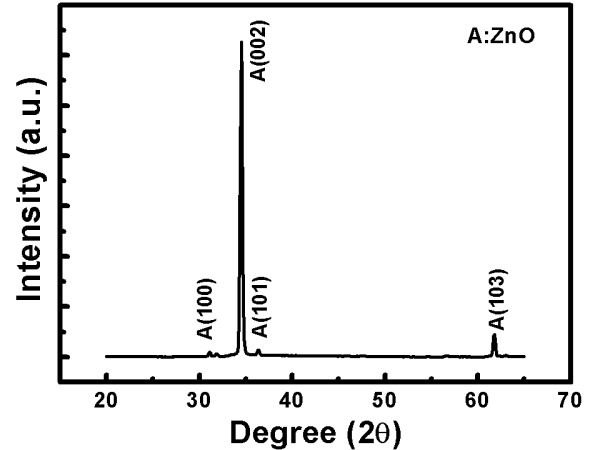
HR-XRD spectra for the non-doped ZnO nanostructures.

**Figure 2:**
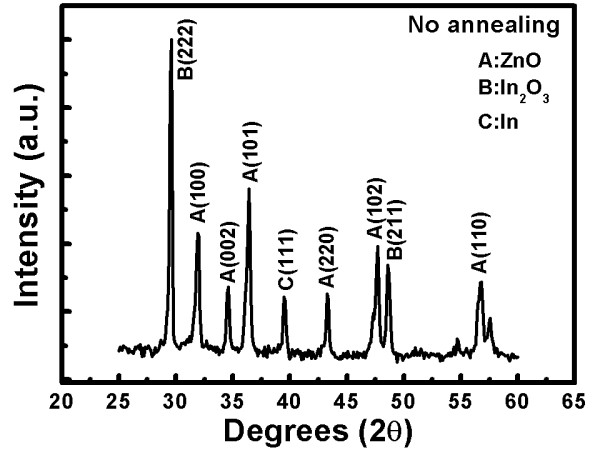
HR-XRD spectra for indium doping in the ZnO nanostructures.

Figure 1 shows a diffraction peak coinciding with the wurtzite structure of ZnO. The strong (002) diffraction peak can be compared to the other peaks such as (101), (102), and (103) that appear to have a weak intensity. Owing to the strong and noticeable diffraction peak, we infer that all the ZnO nanostructures grow along the (002) direction, perpendicular to the substrate, and are well crystallized. From Figure 2, we learn that the (002) intensity is absolutely lower than that of non-doped indium (Figure 1). Figure 2 also shows the peaks appearing in the Joint Committee on Powder Diffraction Standards (JCPDS) database which shows many peaks such as In, In_2_O_3,_ and ZnO. Besides, in this sample, we put doping indium into ZnO, resulting in many increased diffraction peaks such as In_2_O_3_ (222) and indium (111) which are then checked by the JCPDS database. Therefore, it is confirmed that doping indium into ZnO nanostructures in this experiment is successful.

**Figure 3:**
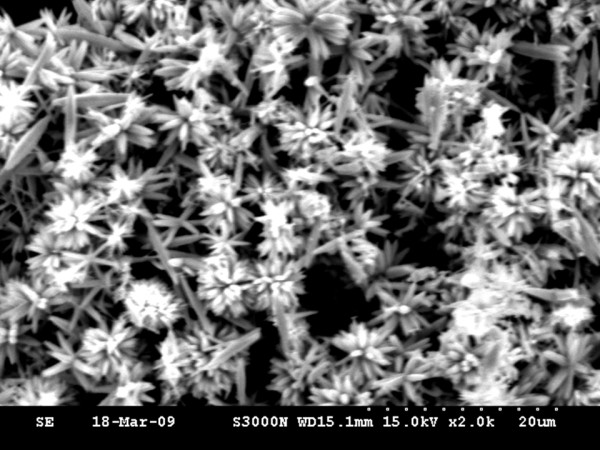
**FE-SEM image.** Image of the surface morphology of the indium-doped ZnO nanostructures after annealing at a temperature of 1,000°C.

**Figure 4:**
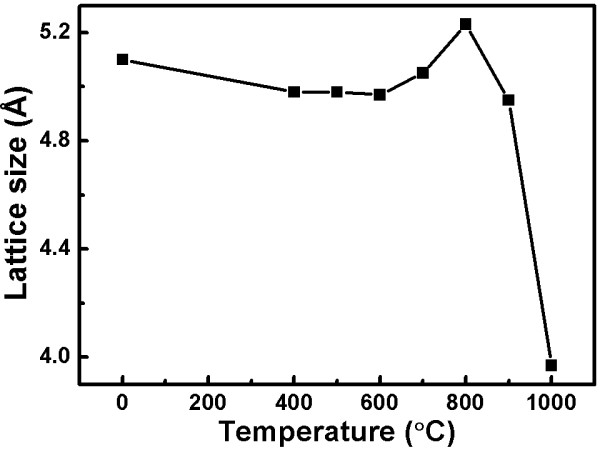
Annealing temperature-dependent lattice size of the indium-doped ZnO nanostructures.

In this study, we observe the samples by the FE-SEM in order to realize the surface structure and the results shown in Figure 3. The elemental analysis or chemical characterization of the ZnO nanostructures by the energy dispersive spectrometer could be estimated. We can observe that the indium element exists which proved that doping indium in the ZnO nanostructure is a success. We can also see the formation of the rod-like morphology of the nanostructures in the SEM image. Most of the nanostructures remain grouped to form a flower-like morphology. The average length of the needle-shaped nanorods ranges from 66, 200, 250, to 400 nm, with an average diameter of 3 μm. We note that the ZnO nanocrystals are obviously shortened and incorporated with the nearby ZnO. In addition, the surface of the ZnO nanostructure diminishes along with increased annealing temperature, and the crystal size can be roughly calculated by [21].

(1) $$\beta_{\mathrm{hkl}}=\frac{k\lambda }{L_{\mathrm{hkl}}\cos\theta }$$

(Scherr equation),

where *β*_*hkl*_ is the value of the FWHM, *k* is the Scherr equation constant for 0.94, *λ* is the XRD diffraction wavelength 1.54 Å, *θ* is the diffraction angle, and *L*_*hkl*_ is the crystal size. We find that the FWHM becomes gradually larger from non-annealing to annealing at 1,000°C which indicates that the ZnO lattice size becomes smaller along with increased annealing temperature, as shown in Figure 4. Inside the crystal, structural changes are caused by different annealing temperatures, and the FWHM of the diffraction peak (002) rises, which is why the crystal size diminishes [22-24].

**Figure 5:**
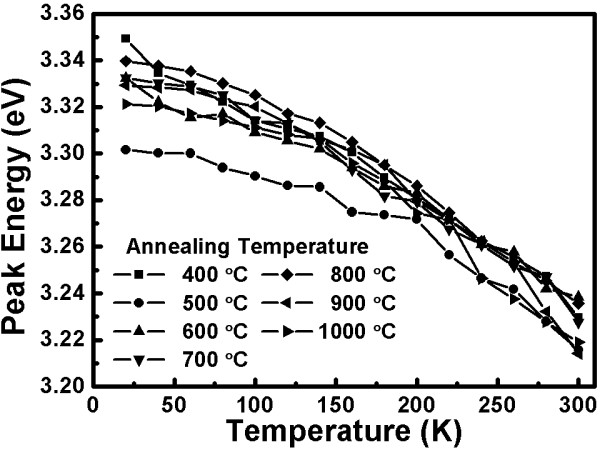
**Temperature dependence of the UV emission peak energy.** For the indium-doped ZnO nanostructures after annealing at different temperatures.

**Figure 6:**
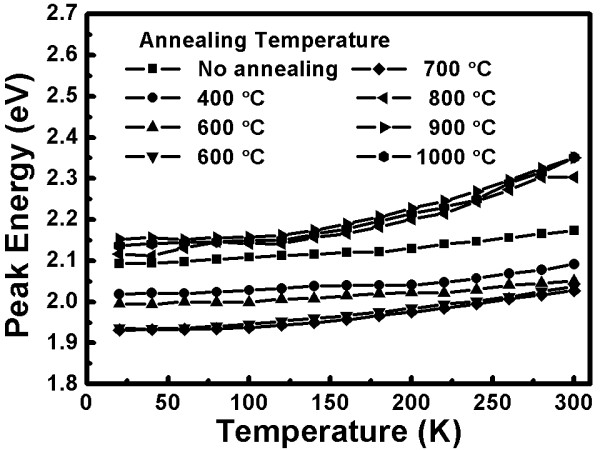
**Temperature dependence of the yellow-orange emission peak energy.** For the indium-doped ZnO nanostructures after annealing at different temperatures.

Under high annealing temperatures, vacancy emissions will lead to a rise in the peak energy and oxygen proceeds to replace the zinc location. This process not only forms O_Zn_ vacancies but also enhances the intensity of vacancy emission in highly oxygenated environments. The bandgap of the semiconductors narrows when the crystal size increases. High-temperature annealing induces more defects inside the crystal and worsens the crystallinity. wavelength and a violet emission at the 405-nm [26] wavelength. In addition, not only are there yellow-orange and violet emissions found in the samples but we also find ultraviolet light of 380 nm and weak far-infrared light of 800 nm. In order to understand the dependence between the PL peak energy and temperature, a Gaussian distribution curve is used to fit the data. Figure 5 shows the temperature dependence of the UV emission peak energy for the indium-doped ZnO nanostructures after annealing at different temperatures. In Figure 5, it can be seen that due to the doping, we find a redshift of the UV emission peak, which can be attributed to the narrowing bandgap energy. As the measurement temperature increases, the peak energy of the UV emission decreases. This phenomenon is the same as that between the UV and violet emissions. Figure 6 shows the temperature dependence of the yellow-orange emission peak energy for the indium-doped ZnO nanostructures after annealing at different temperatures. Nevertheless, based on the contrast in Figure 6, we find that the peak energy of the non-annealed samples is 2.1 eV at a low temperature (20K); however, the peak energy moves higher (2.18 eV) when the measurement temperature increases. This phenomenon is called a blueshift and is shown in all of the other samples produced with different annealing temperatures. In fact, the peak energy increases along with varying measurement temperatures. Unlike the redshift shown with the common semiconductors, the ZnO nanostructures in this experiment reveal different blueshift action. It is generally accepted that the redshift can be attributed to the temperature induced bandgap shrinkage [27] and the changing of the electron-photon interrelation, which can be predicted by the well-known Varshni relation. The reasons for the blueshift of the ZnO nanostructures are presented below.

**Figure 7:**
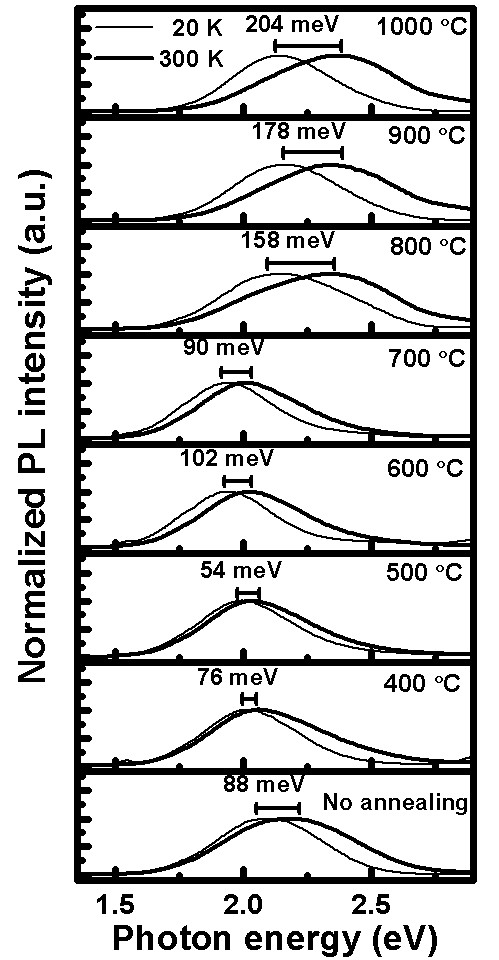
**Normalized PL spectra.** Spectra of the indium-doped ZnO nanostructures after annealing at different temperatures measured at temperatures of 20 and 300 K. The scale bars both show the blueshift energy change of the yellow-orange emission peak energy for the indium-doped ZnO nanostructures after annealing at different temperatures, respectively.

Regardless of whether the samples undergo annealing or not, the yellow-orange emission caused by the excess oxygen vacancies in the ZnO all show the blueshift phenomenon, which is more obvious with increasing temperature, as shown in Figure 7. In other words, the PL intensity is related to the photon energy. It can be noticed that blueshift phenomenon is shown for the samples produced at different annealing temperatures and with the measurement temperature moving from low to high. The two curves of Figure 7 present the normalized PL spectra of the indium doping ZnO nanostructure after annealing at different temperatures measured at temperatures of 20K and 300K, respectively. The non-annealed sample shows an 88-meV blueshift from low to high temperatures. This is similar to the samples annealed at 700°C (90 meV) and 1,000°C (204 meV). The breadth of the photon energy is compared for 20 to 300 K, and the samples are arranged from non-annealing to annealing at 1,000°C. They show first narrowing and then subsequent widening.

**Figure 8:**
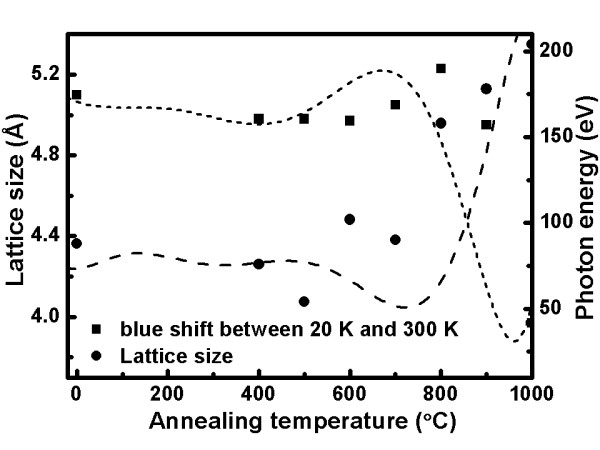
**Annealing temperature-dependent lattice size and blueshift energy change.** Lattice size and blueshift energy change of the indium-doped ZnO nanostructures after annealing at different temperatures.

Figure 8 shows the relationship between the lattice size and blueshift action of the photon energy for different annealing temperatures. From this, we can see that the bandgap of the semiconductor and the crystal size are related. With increasing crystal size, the bandgap of the semiconductor narrows. Therefore, we can see that the bandgap of a semiconductor broadly causes the free electronic transmit through the wider energy level than before which influences the blueshift. Moreover, in this figure, the lattice size and the blueshift are compared to show the relation of the photon energy with the different annealing temperature. Potential reasons for the blueshift to occur are that ZnO decomposes with excess oxygen or there is an interval between the zinc and oxygen. This phenomenon causes vacancies on the surface and lattice shrinkage which then changes the electronic-phonon interrelationship. From past studies, we find that the reason for the blueshift is the luminescence of vacancy [9]. We learn that the samples annealed at 1,000°C have the highest PL intensity, and the intensity rises as the measurement temperature decreases, resulting from the formation of freeze-out phonons.

It is demonstrated that there are two paths for ZnO vacancy emission resulting in strengthened peak energy when the measurement-temperature increases (blue-shift). The first path occurs as low temperature free electrons transit from the conduction band to the Zn_i_ level, then combine with a hole in the oxygen vacancy. Another path is that once free electrons attain enough energy to transit from the conduction band to the center of the vacancies under high temperature and also combine with a hole, resulting in green emission. Therefore, free electrons under high temperatures have more energy to transit the bandgap than under low temperatures which induces the blue-shift action. The experimental results reveal that the carrier-transport process is essentially attributed to the both emissions which are the luminescence of vacancy.

## Conclusions

It is demonstrated that there are two paths for ZnO vacancy emission resulting in strengthened peak energy when the measurement temperature increases (blueshift). The first path occurs as low-temperature free-electron transit from the conduction band to the Zn_i_ level and then combines with a hole in the oxygen vacancy. Another path is that once free electrons attain enough energy to transit from the conduction band to the center of the vacancies under high temperature and also combine with a hole, resulting in green emission. Therefore, free electrons under high temperatures have more energy to transit the bandgap than under low temperatures which induces the blueshift action. The experimental results reveal that the carrier-transport process is essentially attributed to both emissions which are the luminescence of vacancy.

## Abbreviations

FE-SEM, Field-emission scanning electron microscopy; HR-XRD, High-resolution X-ray diffraction; JCPDS, Joint Committee on Powder Diffraction Standards; PL, Photoluminescence.

## Competing interests

The authors declare that they have no competing interests.

## Authors’ contributions

JCW designed and carried out the experiments, statistical analysis, and participated in preparing the draft of the manuscript. YTL and FCC analyzed the data. HIC and CHF offered the technical support. CYT wrote, conceived, and designed the experiments. CYT and CHF contributed to the use of the experimental HR-XRD, FE-SEM, and PL facilities. TEN supervised the research and revised the manuscript. All authors discussed the results, contributed to the manuscript text, and commented on the manuscript. All authors read and approved the final manuscript.
